# A Summer Course in Cancer for High School Students – An Update on Lessons Taught and Lessons Learned

**DOI:** 10.21203/rs.3.rs-4632044/v1

**Published:** 2024-07-22

**Authors:** Xzaviar K. Solone, Siddhi Chitre, Laura Falceto Font, Kimberly N. Espinoza Pereira, Kathryn Stofer, Dietmar W Siemann

**Affiliations:** 1 Department of Molecular Genetics and Microbiology, University of Florida, Gainesville FL, United States; 2 Department of Gastroenterology, University of Florida, Gainesville FL, United States; 3 Department of Neurosurgery, University of Florida, Gainesville FL, United States; 4 Department of Hematology & Oncology, University of Florida, Gainesville FL, United States; 5 Department of Agricultural Education and Communication, University of Florida, Gainesville, FL, United States; 6 Department of Radiation Oncology, Gainesville, FL, USA.; 7 University of Florida Health Cancer Center, Gainesville, FL, United States

## Abstract

**Background:**

Previous graduate students and postdoctoral associates from the University of Florida Health Cancer Center, in partnership with the University of Florida Student Science Training Program, implemented a cooperative learning curriculum, providing high school students with a broad overview of cancer topics over six weeks over the summer. However, the ongoing shift in education and training delivery initially necessitated by the COVID-19 pandemic has given rise to many discussions surrounding student autonomy and satisfaction. Furthermore, adapting hybrid and distance learning styles has notably influenced student-led collaboration and critical thinking skills. Here, we report on an update of experiences gleaned from the modified curriculum of this course to accommodate hybrid and cooperative teaching.

**Methods:**

This pre-post longitudinal observational study evaluated modifications to a cancer biology and therapeutics course. Student performance was assessed using surveys administered before and after the course to determine effectiveness.

**Results:**

Student performance tracked over a 7-year period indicated improved cumulative grade averages after modifying the previous curriculum. Post-assessment analysis revealed significant improvements in student benchmark understanding, notably in their ability to define cancer in one sentence (p = 0.0407), identify cancer therapies (p = 0.0040), and recognize cancer hallmarks (p < 0.0001). An increased trend in median response to the likelihood of pursuing cancer research (p = 0.8793) and the possibility of pursuing cancer research (p = 0.4874) were also observed, although not statistically significant. Moreover, feedback from participating students indicated that “*the educational activities at the end of class (e.g., escape room, case studies)*” and “*learning about cancer and getting to work in groups…*” the curriculum fostered a positive educational learning environment.

**Conclusion:**

Students generally held a positive perception of the course. Post-assessment analysis revealed decreasing trends in students’ perception of course difficulty compared to their expectations in the pre-assessment. Constructive feedback centered around fostering collaborative environments, with an observed increase in learner autonomy towards the end of the course, as evidenced by students’ growing comfort in leading group case studies and conducting research on topics. We hope that future course directors engage actively and incorporate practical clinical examples for students, especially when introducing or discussing complex issues like cancer.

## Introduction

The University of Florida Student Science Training Program (UF-SSTP) is a seven-week residential research program for high-achieving students entering their senior year of high school. Under the mentorship of faculty members, students actively participate in ongoing research projects for 30 hours per week, gaining hands-on experience in current research topics. Celebrating its 65th consecutive year, the UF-SSTP has a long-standing tradition of fostering interpersonal, leadership, professional communication, and organizational skills in its participants. With over 5,000 academically talented students worldwide having completed this rigorous summer residential research program since its inception in 1959, the UF-SSTP provides invaluable opportunities for young scholars to excel. Furthermore, they enroll in UF honors seminar classes created and organized by graduate students and postdoctoral associates to further enhance their academic knowledge and skills.

Since 2011, graduate students and postdoctoral associates at the University of Florida Health Cancer Center (UFHCC) have coordinated a “Cancer Biology and Therapeutics” course. This course has been a transformative experience for lecturers and high school students alike, providing teaching experience to the former while enriching the latter’s knowledge of cancer. In a previous publication by former instructors of this course [1], the instructors tracked students’ performance and discussed the changes over five years. During this period, they observed incremental improvements in cumulative grade point averages. Moreover, the instructors assessed students’ knowledge before and after the course and observed significant increases in understanding benchmarks, particularly in basic cancer knowledge and potential therapeutic options.

Recent studies have indicated that the COVID-19 pandemic has significantly impacted medical education, specifically learning and teaching styles, although the long-term implications are still being debated [2, 3]. The pandemic prompted students and instructors to adapt to remote learning. However, blended cooperative learning environments increase academic satisfaction while promoting student autonomy [4, 5]. Therefore, we aimed to devise a cancer biology curriculum suitable for in-person and remote learning while incorporating cooperative learning methodologies to cultivate student autonomy, an essential attribute for aspiring scientists.

Here, we provide an update on monitoring student progress amid the shift from COVID-19-associated limitations to resuming in-person lectures. We present data to assess lessons learned after course modifications for in-person courses. Our approach hybrids both didactic learning modules with cooperative learning strategies to educate students on recent cancer advancements [6]. Additionally, we exposed students to various career paths through an interactive panel discussion encompassing fields such as epidemiology, law, consulting, biomedical sciences, engineering, physics, medicine, and academia.

## Methods

### Meeting Time and Style

The course convened eleven times on Tuesdays and Thursdays, with each session lasting two hours, totaling 22 contact hours [Table 1]. The class size ranged from 10 to 14 students. Each lecture was divided into two sections: section 1 consisted of prepared lectures, during which students were encouraged to complete follow-along worksheets for engagement and study purposes. In section 2, students engaged in team-based group activities that applied lecture material, including case studies and group assignments. In-lecture assignments were collected, graded, and returned. Students demonstrated their understanding of cancer’s clinical impact through conceptual case studies. They underwent assessment for learning retention via a final exam consisting of multiple-choice, short-answer, and essay questions.

### Student Learning Outcomes

The assessment of learning benchmarks spread across four modules was conducted as follows: 1) Students were evaluated on their ability to develop a solid understanding of basic cancer biology terminology, including terms such as cancer, tumor, oncogene, and tumor suppressor. 2) Student assessment involved exploring the fundamental characteristics of cancer, known as hallmarks, and gaining insights into how cancer cells behave differently from normal cells. 3) Students were tasked with investigating the factors and mechanisms contributing to the development and transformation of cancer cells. 4) Student evaluation included the role of epigenetic regulation in cancer. 5) Students’ understanding of the relationship between the immune system and cancer was assessed, exploring how immune responses can impact disease progression. 6) Assessment involved the influence of the microbiome on cancer prognosis and examining how the diverse gut microbiome in our bodies can affect cancer outcomes. 7) Evaluation included students gaining knowledge about different types of cancer therapies, both traditional and innovative, and understanding their mechanisms of action in treating cancer. 8) Students were exposed to different career options in cancer. Further details about student learning objectives and assessment methods can be found in Table 2.

### Course Lecture Design

The course incorporated several key cancer hallmarks [6] structured into four modules to provide smaller, more manageable units. This approach facilitated a more cohesive and interconnected learning experience, allowing students to build upon and establish connections between concepts as they progressed through each module. Each module was divided into two lectures.

#### Module 1: Molecular Tumor Biology and Overview of Cancer Hallmarks

Module 1 provided a foundational understanding of the course and necessary background information on cancer hallmarks. The topics in lecture one (Introduction and Central Dogma) included DNA structure and synthesis, transcription, protein structure, protein translation, and mutations. To facilitate the team-based group activity, the students were divided into two groups to translate genetic codes. Lecture 2 (Cancer Hallmarks) discussed genomic instability, malignant transformation, oncogenes and tumor suppressors, angiogenic signaling, and cancer metastasis and invasion. The group learning activity comprised two case studies with the following objectives: understanding how to research EGFR and KRAS inhibitors, interpreting survival graphs, comprehending CT and PET scans, and understanding mechanisms of drug resistance.

#### Module 2: Tumor Suppressors, Oncogenes, and Epigenetic Regulation in Cancer

This module was based on the following hallmarks: genomic instability and mutation, evading growth suppressors, sustaining proliferative signaling, and non-mutation epigenetic reprogramming [6]. This module was divided into two parts. The first lecture introduced common mutations observed in cancer, followed by how these mutations contribute to the molecular regulation and role of oncogenes and tumor suppressor genes. The first lecture was followed by a case study covering cancer patient diagnosis & treatment. Each case study had a prompt that required students to think critically and engage with their peers to come up with an answer. The second lecture focused on epigenetics in cancer, highlighting what epigenetics is, critical regulators of epigenetic marks, and the functional consequence of deregulated epigenetic marks in cancer, such as DNA methylation on gene expression. An interesting case study with a real-world scenario in a cancer setting followed this lecture. Case studies proceeded the lecture to introduce how to identify and clinically target dysregulated epigenetic regulators.

#### Module 3: Cancer Immunology and Immunotherapies for Cancer

Cancer Immunology and Immunotherapies for Cancer was the third of four modules in the course and was based on the “Avoiding immune destruction” hallmark [6]. This section was divided into two lectures: “Introduction to Immunology” and “Immunotherapies for Cancer”. The first lecture provided background on the fundamentals of immunology and the relationship between the immune system and cancer. The second lecture, “Immunotherapies for Cancer,” was to provide an understanding of how we can exploit our immune system to create therapies against cancer. Following lecture 1, students engaged in a peer-led case study assignment to identify responses to novel immunotherapy. After lecture 2, students were divided into two teams and participated in a competitive exercise using open notes to assess their comprehensive knowledge of the two lectures.

#### Module 4: Microbiome’s Impact on Cancer Prognosis and Treatment

Module 4 was based on incorporating hallmark “polymorphic microbiome” [6] and therapeutic options. The module was split into two lectures: Microbiome in Cancer and Therapy and the Microbiome. The first lecture introduced the human gut microbiome, microbial dysbiosis, bacteria’s role in cancer, and the importance of a healthy lifestyle to maintain a healthy gut microbiome. The students engaged in interactive learning experiences, such as the Human Gut Game (HGG) [7], a group-based activity that simulates the complexities of the human gut microbiome, allowing them to explore how changes in microbial populations can affect health outcomes, following lecture 1. The second lecture broadened students’ understanding of cancer treatment strategies beyond conventional therapies (i.e., surgery, radiation, chemotherapy). Subsequently, the lecture transitioned to the limitations of those therapies and how the microbiome can be harnessed as a complementary treatment strategy for cancer. For the group learning session, students played an interactive game, “Mystery Box”, that quizzes the understanding of the lecture. Students formed two teams, and each answered 10 questions related to the topic taught during the lecture.

### Case Study Assignment

Students were divided into two cohorts, formed through a random selection process. The students were provided with a rubric outlining the following prompts: Prompt 1: Present the findings related to the development, efficacy, safety, and culmination of a novel drug candidate for glioblastoma in clinical phase 4 trials. Prompt 2: Demonstrate the process of developing a novel diagnostic method for Chronic Myeloid Leukemia (CML), from hypothesis formulation to its implementation in clinical practice. Both cohorts were required to incorporate elements of *in vitro* and *in vivo* studies, team collaboration, data analysis and interpretation, clinical trial designs, and clinical implementation and evaluation. Students’ presentations were to be 15 minutes, followed by 5 minutes of questions by the audience and instructors.

### Escape Room Review

Students participated in examining seven multipart interactive challenges, utilizing their lecture notes to apply a comprehensive understanding of the course material (additional file 1). The learning objectives (additional file 2) encompass foundational knowledge of cancer hallmarks. Additionally, students could participate in a case study aimed at the practical application of the principles governing cancer hallmarks (additional file 3)

### Panel Discussion

Students were assessed to gauge their current career interests, and the panelists were selected based on students’ feedback. The career panel was divided into two parts. The first part was a brief 10-minute presentation about potential careers in STEM given by one of the course instructors. The second and central part was a moderated panel discussion between the different STEM professionals and the high school students. Panelists with expertise in various fields, including engineering, physics, biomedical sciences and research, medical practice, and venture capital and consulting, were invited to participate in the cancer-centric panel discussion.

### Final Assessment

The comprehensive assessment was an online timed examination, allowing only a single attempt, with a duration of 95 minutes. It encompassed 20 questions, divided into four modules, each containing five questions. These questions were structured to include three multiple-choice questions, one short-answer question, and one essay question. Exam questions are included (additional file 4).

## Results

### Cumulative Grade Averages

Trends in students’ final grade averages from 2017 to 2023 are depicted, with individual grade point averages tracked. Cumulative grade averages for 2017–2018 were collected as previously described [1]. The grades presented in 2021 were used as a point of reference, reflecting the modifications made to the original course curriculum for virtual learning. Data for 2022 and 2023 represent grade point averages following additional adjustments to the hybrid curriculum. Statistical analysis using students’ t-tests revealed no significant deviation in student grade averages between 2017 and 2021. However, statistical improvements were observed from 2021 to 2022 (p = 0.0284) and 2023 (0.0372). Each data point corresponds to the grade point average of an individual student.

### Pre and Post Questionnaire

On the first day of the course, students were evaluated on their baseline knowledge of cancer-related topics and their expectations for the course. The assessment was adopted as previously described [1], lasted 5 minutes, and included questions on cancer knowledge and hallmarks, treatment options, and their research interest. The students were re-evaluated on their knowledge on the final day of class. Figure 2 presents the distribution of responses for each question.

#### Define cancer in one sentence.

The students’ responses from both pre-assessment and post-assessment were recorded, pooled, and randomized. Instructors then assigned a numerical grade to each response, ranging from 1 to 10. The mean score increased from 4.9 to 6.6 throughout the course. Statistical analysis using a paired student’s t-test revealed a significant difference in scores (p = 0.0407).

#### Name as many cancer therapies as you can.

Similar recorded therapies were counted only once, and vague answers (e.g., “*drugs*”) were assigned as 0. The mean number of therapies listed increased from 1.7 during the pre-assessment to 3.9 during the post-assessment. Statistical analysis using a paired student’s t-test indicated a significant difference in the results (p = 0.0040).

#### Name as many hallmarks of cancer as you can.

Similar hallmarks were recorded once, and blank responses were assigned a value of 0. Students’ average recollection of cancer hallmarks significantly increased from 0 to 3.8. Statistical analysis using a paired student’s t-test revealed a highly significant difference in the results (p < 0.0001).

#### How close are scientists close to curing cancer?

Students were asked to rate how close scientists were to curing cancer on a scale of 1 to 10 (with 10 indicating high likelihood). The average response scale remained unchanged, with a pre-assessment score of 5.2 and a post-assessment score of 5.2. Statistical analysis using a paired student’s t-test indicated these results were insignificant (p > 0.9999).

#### How likely are you to pursue research?

On a scale of 1 to 10 (with 10 indicating high likelihood), students were asked to rate how likely they were to pursue a research-focused career. The average response scale increased, with a pre-assessment score of 6.5 and a post-assessment score of 6.7. However, statistical analysis using a paired student’s t-test indicated that these results were statistically insignificant (p > 0.8793).

#### How likely are you to pursue cancer research?

Students were asked to rate, on a scale of 1 to 10 (with 10 indicating high likelihood), how likely they were to pursue a career focused on cancer research. The average response scale increased, with a pre-assessment score of 4.9 and a post-assessment score of 5.6. However, statistical analysis using a paired student’s t-test indicated these results were insignificant (p > 0.4874).

#### How difficult do you expect this class to be vs how difficult was this class?

During the pre-assessment, students were asked to rate, on a scale of 1 to 10 (with 10 indicating high likelihood), how difficult they expected the course to be. During the post-assessment, the students were asked to rate how difficult the course had appeared to them. The average response scale decreased, with a pre-assessment score of 6.6 and a post-assessment score of 5.5. However, statistical analysis using a paired student’s t-test indicated these results were insignificant (p > 0.1538).

### Student Evaluation of Course Design

Students received a post-course evaluation from the SSTP program director, which included rating items on a scale of 1 to 5. These items were: 1) Overall, this course provided a valuable educational experience. 2) Course activities and assignments enhanced my ability to analyze, solve problems, and/or think critically. 3) The course content (e.g., readings, activities, assignments) was relevant and useful. Students’ responses were recorded, as depicted in Figure 3.

## Discussion

### Revision considerations for hybrid learning

The UF-SSTP program is an immersive experience for high school students interested in research. Our group designed and implemented a 7-week summer course on the fundamentals of cancer biology, focused on cancer hallmarks. Previous instructors of this course reported steady trends in cumulative grade averages over 5 years and increased basic cancer knowledge. However, this study was published before the COVID-19 pandemic, and therefore, new aspects need to be considered to optimize student learning outcomes post-pandemic. Here, we provided an updated study on teaching methods and student learning outcomes in this summer course on cancer biology after the COVID-19 pandemic.

In 2021, the curriculum underwent revision to accommodate hybrid learning. We partnered with the UF Center for Precollegiate Education and Training (CPET) pre-scholars program to accommodate virtual learning standards and offer a curriculum that could crosslink Florida learning standards with cancer biology. Specifically, we wanted to maintain course rigor while retaining student autonomy and collaboration. The class convened online for eight sessions, each lasting two hours. Consequently, the curriculum was adapted with the following considerations: adjusting the course duration, addressing technical constraints associated with the “Zoom” platform, balancing student autonomy with peer collaboration, upholding the academic rigor of the content despite the absence of nonverbal cues from students, and restructuring the contents of cancer hallmarks [8, 9] into manageable, foundational units. To address these considerations, we implemented “lecture workshops,” that is, didactic lectures followed by breakout room case studies to reinforce lecture material. We also implemented follow-along lecture worksheets and frequently asked questions to engage students throughout the lecture.

Based on prior student feedback, we further revised this course in 2022 to broaden the cancer hallmarks and include more clinical applications. We incorporated cancer immunology and focused on incorporating patient data into the student-led workshops. We also provided additional online resource links to support their analysis. For instance, one case study tasked students with identifying two major types of lung cancer and determining which type is most commonly associated with cigarette smoking. Moreover, they were asked to identify the top three most common mutations for each type and classify whether each mutation is a tumor suppressor gene (TSG) or an oncogene. In this case study, we used clinical data provided by The Cancer Genome Atlas (TCGA) to reinforce the concepts of oncogenes and tumor suppressors. As a learning benchmark, students were expected to determine if their “mock” patient has an oncogenic driver or depletion of a tumor suppressor.

**Figure 1.**
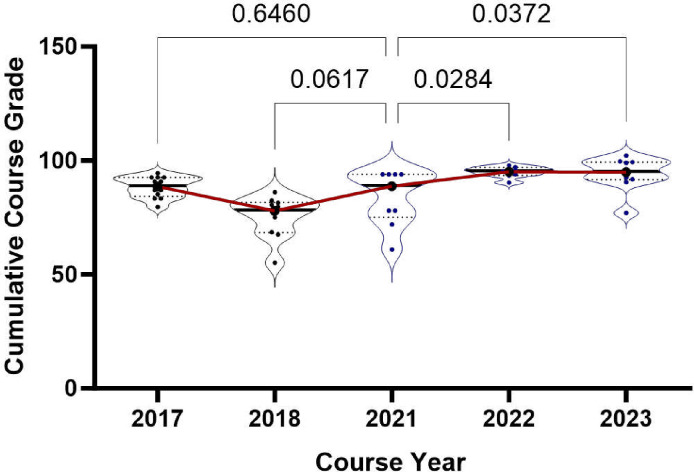
Cumulative Course Performance: Demonstrates the comparison of cumulative exam performance over different periods. Each data point represents an individual’s grade-point average. The exams conducted in 2017–2018 were administered before the revision, while the 2021 exam was conducted virtually after the revision. The exams from 2022–2023 were adjusted based on feedback received.

In 2022, the cancer hallmarks were revised to include “polymorphic microbiomes” and “non-mutation epigenetic reprogramming” [6]. We further revised the curriculum to incorporate these hallmarks into modules, including cancer immunology from the prior year. This speaks to the versatility of the curriculum style, as we saw steady cumulative grade averages between the 2022 and 2023 cohorts (Fig. 1) despite introducing a more rigorous curriculum. In the following sections, we discuss and interpret the pre-post longitudinal observational study results and dive deeper into each course component. Our main objective is to provide guidelines on how we delivered a course on the fundamentals of cancer biology.

### Evaluation of Student Knowledge Comprehension

The final grade averages served as a comparative assessment between years. We included 2017 and 2018 as a reference point for the original curriculum. We cannot account for educational background differences among cohorts. However, students are generally selected from similar regions each year. We altered the curriculum in 2021 to comply with remote learning restrictions. When the restrictions were lifted, we adopted the same approach for future cohort installments (2022 and 2023), and we observed steady rigor in the grades tracked. We also observed improvement in the minimum grade average of 61 to 90.35 in 2022 and 76.98 in 2023 (Table 3). We believe adding follow-along assignments and student-led cooperative group assignments improved students’ autonomy and comfort with the subject area.

**Figure 2:**
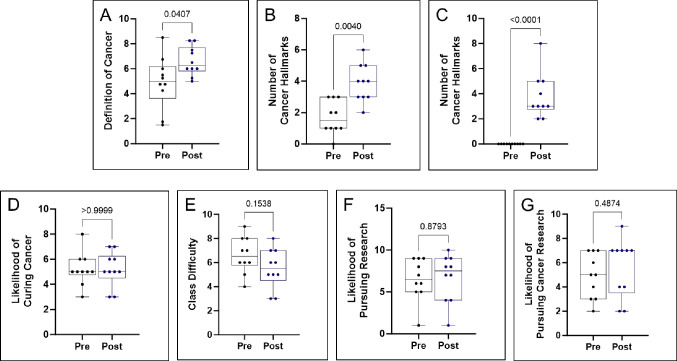
Results of the Pre-and Post-Assessment. (**A**) Definition of cancer: Students’ responses to defining cancer in one sentence, with scores ranging from 1 to 10. (**B**) Cancer therapies: Students’ responses naming cancer therapies. (**C**) Hallmarks of cancer: Students’ responses naming hallmarks of cancer. (**D**) Likelihood of curing cancer: Students’ ratings on a scale of 1 to 10 on the likelihood of scientists curing cancer. (**E**) Likelihood of pursuing research: Students’ ratings on a scale of 1 to 10 on the likelihood of pursuing a research-focused career. (**F**) Likelihood of pursuing cancer research: Students’ ratings on a scale of 1 to 10 on the likelihood of pursuing a career focused on cancer research. (**G**) Perceived difficulty of the course: Students’ ratings on a scale of 1 to 10 on the expected difficulty vs. actual difficulty of the course.

When we asked students to define cancer in one sentence, we observed a significant increase in the quality of the responses in the post-course assessment compared to the pre-assessment (Fig. 2A). For instance, general responses submitted for the pre-assessment included “*a foreign body that can spread throughout the body*” and “*cell mutation in the human body*.” In contrast, in the post-assessment, we observed higher-quality responses such as “*a mutation in a cell that leads to the uncontrolled dividing and spreading of malignant cells.*” We also noticed that some students incorporated cancer hallmarks and terms (“*defect of cells resulting in oncogenes that multiply quickly, spread throughout the body, and cause tumors*”) in the general responses after the course, which indicated that students retained general information throughout the course. Moreover, students also demonstrated the ability to connect key concepts over the duration of the course. For example, some students recognized the link between genomic instability and the potential for cancer-associated antigen presentation, recognizing avenues for targeted therapy or immunotherapy.

We next asked students to name as many cancer treatments as they could (Fig. 2B). During the pre-assessment, a lot of the answers were traditional therapies such as “*radiation*,” “*chemotherapy*,” and “*surgery.*” In the post-assessment, the variety of responses significantly improved to include both standard therapies as well as more precision-based therapies such as “*immunotherapies (CAR T cells)*,” “*gene therapy,*” “*hormone therapy,*” “*fecal microbiota transplant (FMT),*” and “*bone marrow transplants,*” among others. Our focus throughout the course was to increase exposure to targeted therapies and novel therapies not commonly discussed, for instance, “*adoptive cell therapy*” and “*small tyrosine-kinase inhibitors*.” The interactive group learning assignments covered novel therapeutic approaches, likely aiding students’ high retention of the course material.

The most remarkable findings emerged when students were asked to identify as many cancer hallmarks as they could (Fig. 2C). Initially, during the pre-assessment, responses ranged from “*What is a hallmark?*” and “*not sure*” to some leaving the question unanswered. However, following the course, there was a statistically significant increase in the quantity and quality of the responses, exceeding our expectations. For instance, one student provided a summary list including “*sustaining proliferative growth, metastasis, tumor-promoting inflammation, developing immune escape, senescent cells, dysregulated cell metabolism, resisting cell death, genome mutations,*” and another answered “*angiogenesis, cell proliferation, evading cell death, polymorphic biomes, irregular cellular regulation.*” Notably, the students correctly identified cancer hallmarks within the allotted time.

Another notable trend between pre-assessment responses and post-assessment responses is the likelihood of pursuing research (Fig. 2D), specifically cancer research (Fig. 2E). Although no statistical significance could be observed throughout the course, there was a noticeable upward trend in median responses after the course ended. Furthermore, when assessing the course difficulty responses, the data indicate that some students did not feel like the course was as challenging as initially thought (Fig. 2G). However, the responses from that survey were non-statistically different.

**Figure 3:**
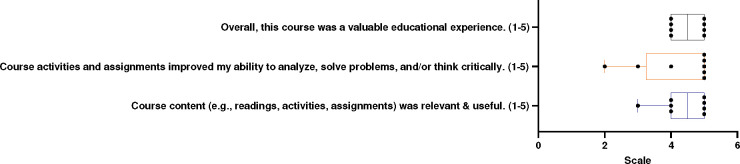
Student Evaluation of Course Design Student evaluation of course rigor. On a scale of 1 through 5, students were asked to evaluate their educational experience, their improved ability to solve problems and critically think, and the course content. Each point represents a student’s response.

The SSTP program director also gathered feedback from students regarding the course difficulty and design (Fig. 3). Students were asked to rate three prompts on a scale of 1–5, where 1 indicated “*strongly disagree*” and 5 indicated “*strongly agree*.” To the first prompt (overall, this course was a valuable educational experience), 50 % of students answered 4, and the other 50 % gave a score of 5, indicating the students found the course to be of educational value. This was coupled with additional feedback, with one student stating: “*The case studies were a lot of fun and provoked critical thinking in a way I’ve never experienced*”.

The second prompt interrogated if the course activities and assignments improved the students’ ability to analyze, solve problems, and/or think critically. While most students (62.5 %) gave a rating of 5, one student gave a response of 2, another gave a score of 3, and the other gave a score of 4. Additional feedback highlighted the effectiveness of educational activities such as escape rooms and case studies, with responses like: “*The educational activities we did at the end of class (e.g., escape room, case studies)*” and “*Learning about cancer and getting to work in groups and play games*” further reinforcing the curriculum style. The final prompt asked whether the course content (e.g., readings, activities, assignments) was relevant and valuable. Once again, most students responded with a rating of 5 or 4 (50 % or 37.5, respectively), with only one response rating of 3. Overall, we felt confident that these students benefited from the course and enhanced their learner autonomy.

Students were also encouraged to provide additional comments, and only one did so. The response mentioned, “*The terminology was difficult to fully understand, and the lessons felt a little rushed each time. Adding more assignments would help us understand the information more and apply it*.” Although the course is condensed into 22 contact hours, future curriculum revisions may consider providing optional pre-reading assignments to help students grasp more challenging concepts.

### Curriculum Modules and Content

Module 1 introduced the course’s foundation and reinforced critical biological concepts. These include “central dogma” and “mutation difference leading to disease.” For instance, chromatin translocation of the t (9;22) (q34; q11) (Philadelphia chromosome) comprising the ABL gene and the BCR gene, producing BCR-ABL oncogene is a famous fusion event driving chronic myeloid leukemia (CML). The learning objective was for students to investigate the factors and mechanisms contributing to cancer cell development and transformation, Table 1. The following lecture was to introduce students to all cancer hallmarks. We based this lecture on the recent updates to cancer hallmarks [6]. We expected students to explore the fundamental characteristics of cancer hallmarks and gain insights into how cancer cells behave differently from normal cells. The students were exposed to unfamiliar topics for the student-led group assignment and learned how to research and interpret data. For instance, students needed to research the molecular target for erlotinib (Tarceva) before analyzing the Kaplan Meier (KM) survival plot.

A critical hallmark of cancer is genomic instability and mutation. The second lecture focused on understanding what DNA mutations were and teaching students that multiple types of mutations can have varying effects depending on the gene mutated. Students were taught the difference between a Tumor Suppressor and an Oncogene, one of the fundamental lessons of cancer biology that will help set the stage for greater comprehension of the rest of the course. Two hallmark genes (p53 and KRAS) were discussed in depth to provide an example of one tumor suppressor and oncogene. Moreover, students engaged in two case studies covering cancer patient diagnoses & treatment to promote a deeper understanding of the topic. Each case study had a prompt that required students to think critically and engage with their peers to come up with an answer. The objective of the lecture and case study activity was to help students investigate the factors and mechanisms contributing to cancer cells’ development and transformation.

Epigenetics is a less common but important concept in cancer. Thus, we wanted to introduce students to an unfamiliar topic. At the beginning of the lecture, students took a survey question to explain, “What makes a neuron different from a muscle cell?” The goal was to help students understand the importance of epigenetics in biology. The idea of epigenetics was explained, though the lecture only focused on DNA methylation for simplicity. The students were then asked to hypothesize the level of DNA methylation on a tumor suppressor gene and an oncogene to assess their comprehension. Following the lecture, the students were then tasked with two short case studies reflecting on actual situations in which DNA methylation was altered in cancer or during famine and had to hypothesize how DNA methylation could change in response to these events. One final case study was presented, in which the whole class had to collaborate to come up with possible treatment options if a patient had a mutation in an enzyme causing DNA methylation or in an enzyme removing DNA methylation. This discussion helped students fully understand the role of DNA methylation, the process of DNA methylation, and how disruption/mutations in this cycle could be relevant in cancer & how to target it for treatment.

The overall learning objective for module 3 was to teach students that the immune system protects us against cancer, but when it fails, cancer can develop. Specifically, it was important for students to understand the relationship between the immune system and cancer and how immune responses can affect the progression of the disease. Important concepts covered in this lecture were the following: the science of immunology and examples of immunity, history of immunology (e.g., smallpox vaccine as an example of how vaccines work), development of immune cells and the immune response (e.g., innate and adaptive immune responses), immune system defenses against cancer, and why the immune system sometimes fails to protect us. The goal of the second lecture, “Immunotherapies for Cancer,” was to provide an understanding of how we can exploit our immune system to create therapies against cancer. Immunotherapy is a growing field that has already improved the standard of care for malignancies such as melanoma [10]. This lecture aimed to help students understand that immunotherapies may not be effective for all cancer patients and that different immunotherapies may be used to treat different cancer types. Concepts covered in this lecture were the tumor microenvironment and the role of immune cells within, neoantigens and mutations, immunosurveillance, the cancer immunity cycle, and types of immunotherapy and how they work against cancer (adoptive cell therapy, CAR T-cells, immune checkpoint inhibitors, and cancer vaccines).

The incorporation of the “polymorphic microbiome” into the Hallmarks of Cancer [6] has illuminated its significant role in intersecting with genetic factors and inflammation for supporting cancer. This growing field presents exciting prospects for advancing our understanding and potentially harnessing the microbiome to enhance cancer prevention and treatment strategies. The primary objective of educating high school students about the microbiome’s role in cancer is to provide them with a foundational comprehension of how the diverse microorganisms within our bodies can significantly influence our overall health. It was crucial to emphasize that even a minor disruption in the gut microbiome can potentially contribute to the development and progression of cancer. Moreover, it endeavors to impart to them an awareness of the significant link between alterations in the microbiome and cancer, elucidating the mechanisms through which specific microorganisms can influence cancer risk. In pursuit of this goal, students engaged in interactive learning experiences, such as the Human Gut Game (HGG) (2), a group-based activity that simulates the complexities of the human gut microbiome, allowing them to explore how changes in microbial populations can affect health outcomes. This educational way encourages critical thinking and exploration of ongoing research, nurturing a sense of curiosity and a passion for lifelong learning in microbiome-cancer interactions while fostering communication skills. Furthermore, the course concluded with a lecture covering treatment strategies available in cancer clinics and the diverse approaches for modulating the microbiome as a potential foundation for treatment.

### Cooperative Learning Assignments- Case Studies, Presentation, and Escape Room

Throughout our course, we introduced students to “mock” clinical case studies that revolved around crucial aspects of cancer research, intending to familiarize them with cooperative learning environments. In order to help students understand the relationship between cancer hallmarks and the clinical practicality of applied and translational sciences, the students were assigned into two groups. The first group presented the findings on the development, efficacy, safety, and culmination of a novel drug candidate for glioblastoma in clinical phase 4 trials. Specific instructions were to form a hypothesis proposing a potential drug target or approach to inhibit tumor growth or enhance treatment response, to include pre-clinical analysis (*in vitro* and *in vivo*), team collaboration (identify interdisciplinary teams for completion of the project), data analysis and interpretation, and to design a clinical trial from phase 1–3 (considering the ethical implications). The second group was tasked with demonstrating the journey of developing a novel diagnostic method for CML, from hypothesis formulation to its implementation in clinical practice. Specifically, they were to hypothesize a specific biomarker or genetic aberration that could serve as a reliable diagnostic marker for early detection of CML. Similarly, the case study must include pre-clinical analysis, team collaboration, data analysis and interpretation, and clinical trial design. These case studies served as a valuable platform for enhancing the students’ presentation and communication skills, fostering teamwork, and applying scientific principles to real-world scenarios. Their presentation encompassed essential elements within their case study. Their presentation adeptly highlighted the translational aspects of the research, bridging pre-clinical findings to clinical applications. Furthermore, they tackled challenges, outlined anticipated clinical outcomes, and thoughtfully addressed the potential for long-term effects, stressing the significance of continuous safety and efficacy monitoring.

The escape room activity fostered students with a comprehensive open-note assessment featuring seven challenging, multistep questions. Additionally, “eliminated” students engaged in a cancer hallmark worksheet covering lecture-discussed hallmarks in a multistep case study format. The escape room aimed to explore mechanisms involved in evading immune destruction, including concepts such as tumor immune profiles (immune inflamed, immune desert, and immune excluded), as well as strategies for evading growth suppressors, genomic instability and mutation, sustaining proliferative signaling, and the impact of the polymorphic microbiome. Emphasis was placed on understanding adaptive and innate immune responses and non-mutational epigenetic reprogramming.

### Panel Discussion

At the end of the course, we also organized a career panel for the students to learn about the wide umbrella of opportunities within the STEM field. We included panelists from a wide range of STEM careers, representing diverse stages in their professional journeys:

- Biomedical Sciences PhD student in the final stage of their PhD
- MD/PhD student in their second year of PhD (2 years of medical school already done)
- Venture capitalist with experience in consulting (PhD in Biomedical Sciences)
- Bioinformatician (PhD in Biomedical Sciences)
- PhD student in Physics focusing on biophysical applications in cancer
- Postdoctoral student in Biomedical Engineering with a focus on CRISPR gene therapy
- Physician and Principal Investigator of a laboratory (MD)
- Radiation Oncology Physician Resident (MD)

First, the panelists introduced themselves, briefly describing their career trajectories. The intention behind this was to familiarize students with key steps and diverse strategies for entering STEM careers. After that, we opened the floor to ask the students questions. Some of the topics and questions that were discussed that can serve for future panel discussions are summarized below:

- Why did you become interested in your career?
- What steps did you take to get to where you are today in your career?
- What are the differences between medical school, graduate school (MS or PhD), and MD/PhD? How are the requirements different?
- Do you enjoy your current role?
- If you could start your career over, would you choose the same career path?
- What advice do you have for high school students planning to go to college and pursue a STEM career in the future?

### Limitations of this study

One important caveat is that we are not comparing online virtual learning from the CPET pre-scholars program in 2019 to the in-person modified course in 2023. The conditions and requirements for student selection were changed in 2021. However, we are tracking graduates across five years to assess if our modifications in 2019 disparaged grading standards when students were allowed to return to in-person learning. Therefore, the grades tracked over time are used to ensure grading standards. Also, this study includes the lack of consistent final exam evaluation between 2017 and 2022, which may have introduced variability in long-term data consistency and instructor changes over the years. Course feedback and cumulative grades from 2019–2022 have been collected, dating before and during course modification. However, cumulative grade point averages were not presented, limiting the comprehensive understanding of student performance. Furthermore, variations in teaching styles due to changes in course instructors could have influenced the results, suggesting the need for consistency in instructional approaches for future studies. Lastly, our pre-post observational study was conducted for one cohort. Future instructors should track responses for long-term and consistent results.

## Conclusion

Overall, our course aimed not only to teach students about different concepts of cancer development and hallmarks but also to encourage students to explore different careers within the STEM field. In our experience, we have found that students at the high school stage have not been exposed to a wide range of STEM careers, leading to many students focusing on a career in medicine as their only option. In this course, we aimed to highlight alternative STEM careers to medicine to help students make informed decisions on their future careers.

In summary, we imparted a summer course on cancer hallmarks, adapting our teaching strategy to the post-COVID-19 era and measuring student learning outcomes. The results indicated that students performed better in the post-course assessment than in the initial evaluation, indicating that students could successfully understand and retain concepts related to diverse cancer hallmarks. Students also demonstrated improved critical thinking and enhanced collaborative and communication skills. We hope our detailed overview of teaching methodologies and outcome assessment approaches can assist fellow instructors in crafting courses in the post-COVID-19 pandemic era. This curriculum can be adapted based on the level and the number of students per class, and it can be modified to cover additional cancer hallmarks.

## Figures and Tables

**Table 1:** Course Schedule and Topics

	Date	Topic	Assignment
1	06/15	Central Dogma	Group TBL Assignment
2	06/20	Cancer Hallmarks	Case Study TBL Assignment
3	06/22	Oncogene and Tumor Suppressor	TBL Assignment
4	06/27	Epigenetics and Cancer	TBL Assignment
5	06/29	Introduction to Immunology	Case Study TBL
6	07/04	No Class	No Class
7	07/06	Immunotherapies for Cancer	Game Choice Activity
8	07/11	Microbiome in Cancer	TBL Assignment
9	07/13	Therapy & the Microbiome	TBL Assignment
10	07/18	Escape Room/Review	Group Project (TBD)
11	07/20	Careers in Science Panel	Final Assessment Opens
12	07/25	End of the Course Celebration	

**Table 2:** Course Alignment Map

Fundamentals of Cancer Biology Alignment Map				
Course Objective	Module Objectives	Resources	Assessments Assignments	
		Required	Required	Optional
Develop a solid understanding of basic cancer biology terminology, including terms like cancer, tumor, oncogene, and tumor suppressor.	Module 1: Molecular Tumor Biology and Overview of Cancer Hallmarks	In class follow along worksheets.	Students will be divided into 2 large groups and deliver a structured presentation based on a predefined case study, focusing on Glioblastoma and Chronic Myeloid Leukemia.	Decoding Cancer Comprehensive Worksheet
		TBLs worksheets.		
Explore the fundamental characteristics of cancer, known as hallmarks, and gain insights into how cancer cells behave differently from normal cells.	Module 2: Tumor Suppressors, Oncogenes, and Epigenetic Regulation in Cancer	In class follow along worksheets.		
		TBLs worksheets.		
Investigate the factors and mechanisms that contribute to the development and transformation of cancer cells.	Module 3: Cancer Immunology and Immunotherapies for Cancer	In class follow along worksheets.		
		TBLs worksheets.		
Learn about the role of epigenetic regulation in cancer.	Module 4: Microbiomes’ Impact on Cancer Prognosis and Treatment	In class follow along worksheets.		
		TBLs worksheets.		
Understand the relationship between the immune system and cancer and how immune responses can affect the progression of the disease.	Final Assessment: Comprehensive Timed Quiz		The Comprehensive Assessment is a single-attempt, time-limited exam lasting 95 minutes and covering 20 questions. Each module consists of 5 questions, including three multiple-choice questions, one short-answer question, and one essay question.	Create a 5 to 10-minute presentation that covers a topic within Cancer Immunology (of your choosing) by the last day of class. This assignment can be done individually or with a group.
Introduce the gut microbiome in healthy and diseased states. Understand the role of pathogenic bacteria in cancer development, gain knowledge about different types of cancer therapies, both traditional and innovative, and understand how they work to treat cancer. Explore different therapeutic options used in the clinic to modulate the gut microbiome.				
Introduce students to different types of cancer therapies, both traditional and innovative, and understand their mechanisms of action in treating cancer.				
Expose students to different career options in cancer.				

**Table 3:** Frequency Distribution and Descriptive Statistics of Cumulative Grade Averages

	2017	2018	2021	2022	2023
	
Total number of values	13	10	9	8	10
Minimum	79.63	55.09	61.00	90.35	76.98
25% Percentile	84.26	68.29	75.00	93.55	91.51
Median	88.89	78.24	89.00	95.40	95.15
75% Percentile	92.59	81.71	94.00	96.88	99.35
Maximum	94.44	86.11	94.00	97.81	102.15
Mean	88.32	75.32	83.78	94.95	94.21
Std. Deviation	4.50	9.24	12.09	2.37	7.17
Std. Error of Mean	1.25	2.92	4.03	0.84	2.27
Lower 95% CI of mean	85.60	68.72	74.48	92.96	89.08
Upper 95% CI of mean	91.04	81.93	93.07	96.93	99.34

## Data Availability

The data supporting the findings of this study are available upon reasonable request from the University of Florida Cancer Center.
